# Combined exosome of adipose-derived mesenchymal stem cell and hyaluronic acid delays early osteoarthritis progression of ovine sheep model: Clinical, radiographic, macroscopic and microscopic evaluation

**DOI:** 10.12688/f1000research.147309.3

**Published:** 2025-02-13

**Authors:** Ludwig Andribert Powantia Pontoh, Jessica Fiolin, Ismail Hadisoebroto Dilogo, Marcel Prasetyo, Radiana Dhewayani Antarianto, Alida Harahap, Angela Jennifer Tantry, Trevino Aristakus Pakasi, Bambang Pontjo Priosoeryanto, Tri Isyani Tungga Dewi

**Affiliations:** 1 Orthopaedic and Traumatology, Faculty of Medicine, Universitas Indonesia, Centra Jakarta, DKI Jakarta, 10430, Indonesia; 2 Doctoral Program of Medical Science, Faculty of Medicine, Universitas Indonesia, Central Jakarta, DKI Jakarta, 10430, Indonesia; 3 Department of Radiology, Faculty of Medicine, Universitas Indonesia, Central Jakarta, DKI Jakarta, 10430, Indonesia; 4 Department of Histology, Faculty of Medicine, Universitas Indonesia, Central Jakarta, DKI Jakarta, 10430, Indonesia; 5 Department of Biology, Faculty of Mathematics and Natural Science, Universitas Indonesia, Depok, West Java, 16424, Indonesia; 6 Department of Community Medicine, Faculty of Medicine, Universitas Indonesia, Central Jakarta, DKI Jakarta, 10430, Indonesia; 7 School of Veterinary Medicine and Biomedical Science, Institut Pertanian Bogor, Bogor, West Java, 16680, Indonesia

**Keywords:** exosome, adipose-derived Mesenchymal Stem Cell (Ad-MSC), early osteoarthritis (OA), clinical lameness score, radiographic OA score, Osteoarthritis Research Society International (OARSI) score

## Abstract

**Background:**

Current treatment of osteoarthritis (OA) mainly focused on treating symptoms. Exosome from Adipose-derived Mesenchymal Stem Cell (Ad-MSC) have been shown to delay degenerative process. This study aimed to investigate the clinical, radiological and histological impact of combined intra-articular (IA) hyaluronic acid (HA) and exosome Ad-MSCs in-vivo using a larger animal model with low-grade OA.

**Methods:**

Eighteen male *Ovies aries* sheep underwent total lateral meniscectomy and conventional radiography was performed to confirm low-grade OA after 6 weeks. The sheep were divided into three groups, Group 1 (G1; n=6) received thrice exosome injections, G2 (n=6) received twice HA injection, and G3 (n=6) received both treatments with a 1-week interval after 10 days of meniscectomy. Clinical evaluations were conducted using the Clinical Lameness Score (CLS), radiographic with X-ray using OA score by Innes et al, while macroscopic evaluation by Osteoarthritis Research Society International (OARSI) scores.

**Results:**

Lameness parameter scored lowest in G3 significantly (2.0±0.0 VS 2.7±0.52 VS 2.7±0.52; p=0.024) at the second month although the overall CLS score did not significantly differ at the 3 ^rd^ month. The best improvement of conventional total OA radiographic score at the 3 ^rd^ month compared to all groups (5.2±1.17 vs 6.3±0.82 vs 6.7±1.03; p=0.053). Macroscopic OARSI evaluation showed no difference (p=0.711).

**Conclusions:**

Combined repeated exosome Ad-MSC and HA IA injection proven to delay OA progression, however longer duration of follow up is required to evaluate its long-term effect.

## Introduction

One of the most common degenerative joint diseases that place a significant socioeconomic burden on society worldwide is knee osteoarthritis (KOA). ^1^ However, no effective treatment can stop osteoarthritis’ (OA) increasing cartilage deterioration. The current cornerstone of OA therapy continues to focus on providing momentary symptomatic alleviation. ^2^ Even though the present knee replacement operation for end-stage OA is regarded as the most successful procedure of the century, many patients continue to weigh the cost, risk, and complications of such significant surgery. ^2^

Research on the chondrogenic potential of mesenchymal stem cells (MSC) has been focused primarily on recent developments in regenerative orthobiologic therapy. ^3^^,^^4^ Recent research, however, indicated that exosomes and other trophic factors were primarily responsible for the potency of MSC. ^5^^–^^7^ Additionally, despite being promising, the use of MSC for OA treatment has several disadvantages, including ethical concerns, genetic instability, immunological reactions to transplanted cells, challenges with mass production and storage, and cost effectiveness. On the other hand, exosomes have many benefits over MSCs, such as a superior safety profile with fewer side effects, a lower immunogenicity reaction, the capacity to penetrate barriers that MSCs cannot, an easier mass production and storage process at a lower cost, and fewer ethical concerns. ^8^^,^^9^

Exosomes are nanosized extra vesicles with 50–150 nm diameter that are secreted by MSCs and contain nucleic acids, functional proteins, and bioactive lipids. Their duties include controlling immune responses, decreasing inflammation, and healing damaged tissues. Exosome isolation from several MSC sources has been described. ^6^ However, compared to bone marrow-derived MSC (BMMSC) and synovial MSC (SMSC) derived exosomes, adipose-derived MSC (ADMSC)-derived exosomes have shown a remarkable potential to stimulate cartilage and bone regeneration. Exosomes and microvesicles produced by ADMSCs can regulate focal adhesion, extracellular matrix (ECM)-receptor interaction, actin cytoskeleton, cAMP, and PI3-Akt signaling pathways, which can correct aberrant osteoblast metabolism and promote cartilage and bone regeneration. ^10^

Hyaluronic acid (HA) is a crucial part of the synovial fluid that cushions and protects joint cartilage. The knee joint’s HA maintains a steady concentration and enough viscosity. Reduced HA concentration brought on by OA exacerbates knee cartilage damage. Further, HA can encourage cell migration and it is advised to administer repeated HA injections for knee joint disease. Additionally, several clinical trials have shown that HA can lessen OA patients’ pain. ^11^^–^^14^

Clinical, radiological, and histopathologic examinations can be used to evaluate the efficacy of combined exosome-HA therapy. To assess the course of OA in animals, several radiological scoring systems, such as those created by Innes et al. in 2004, ^15^ have been devised. While waiting for treatment to take effect, macroscopic analysis can be utilized to determine how badly damaged the cartilage has become. ^16^ Sheep and humans are comparable in size, biomechanics, and joint structure. Because of these similarities, sheep were a useful animal model for studying cartilage macroscopic processes in orthopaedic investigations. ^17^

The clinical impact of combined intraarticular HA and exosome-derived ADMSC in vivo on a bigger animal model with low-grade OA has never been studied so far. This investigation aims to assess the clinical, radiological and macroscopic effects of combined hyaluronic acid and intra-articular ADMSC-derived exosomes in the ovine early OA model.

## Methods

The study protocol was approved by the institutional review board no. KET-932/UN2.F1/ETIK/PPM.00.02/2022. The animal studies were performed after receiving approval from the Institutional Animal Care and Use Committee (IACUC) in the School of Veterinary Medicine and Biomedical Sciences at Institut Pertanian Bogor (IPB) University number 023/KEH/SKE/IX/2022 from the obtained for this work. All procedures and protocols, encompassing the research question, design, and analysis strategy, were executed in adherence to the ARRIVE guidelines.

All animals underwent a one-week adaptation with a vaccination and health check-up protocol. The sheep were kept in six paddocks, 3 in each, with stone yards for resting, and were also fed concentrate, hay, and mineral salt. Ad libitum water was supplied in man-made drinking troughs. Before each medical procedure that require operative procedure, we performed anesthesia to ameliorate any suffering of the animals.

We calculated the sample size using resource equation and the equation showed that a minimum of 5 sheep need to be allocated in each group. Thus, we included a total of 18 *Ovies aries* for this study. Inclusion criteria include males, age ≥ 3 years old, weight 25-30 kg, and skeletal maturity. Exclusion criteria include musculoskeletal abnormalities, death, infection, and cartilage defects prior to meniscectomy.

### Exosome Ad-MSC preparation and characterisation

Cryoprecipitate of secretome AD-MSC from human was collected and kept at -20°C. By submerging the frozen CM container in room temperature water, the frozen CM was defrosted. The CM solution was centrifuged for 15 minutes at 200C and 750×g of speed. After collecting the supernatant, it was centrifuged again for 15 minutes at a speed of 2000×g. After that, the supernatant was gathered and spun for 45 minutes at 10,000×g. After collecting and filtering the supernatant using a 0.2 μm syringe filter, 90 minutes of ultracentrifugation at 4°C at a speed of 100,000×g was carried out. After that, the pellet containing exosome was transferred to a 15 mL falcon tube and the supernatant was discarded. After adding cold D-PBS till the volume reached 5 mL, the mixture was re-dissolved. Subsequently, the exosome were separated into a 1 mL cryovial and stored for a year in either a freezer at -80°C or a cryo-box chiller at -20°C.

Afterwards, exosome was checked for sterility and characterisation using flowcytometry assay which showed positive for CD63 and CD81. Using a Horiba SZ 100z particle size analyser (PSA), which can also determine the suspension sample's molecular weight and zeta potential, the exosome size and distribution were assessed. Zeta potential was performed in triplicate for each experiment at 25°C. The exosome showed mean size of 88.7 nm ± 40 nm standard deviation (SD), zeta potential of -1.4 mV and -10.2 mV and conductivity was between 14.945 mS/cm and 14.982 mS/cm. ^18^

### Meniscectomy induced osteoarthritis procedure

A total of 18 *Ovies aries* sheep received lateral meniscectomy at the stifle joint of the right hind limb following the procedure mentioned in the previous study. ^19^ Two veterinarians performed the meniscectomy procedure. Amoxicillin 5 mg/kg and atropine sulfate 0.15 mg/kg were administered intramuscularly and subcutaneously before the surgery. Ketamine 22 mg/kg and xylazine 0.20 mg/kg were administered intramuscularly for the anesthesia. Ten days after meniscectomy, the dressing was opened, and the wound had perfectly healed, so rehabilitation was started. For two weeks, the ovines were trained to walk for 150 meters on an asphalt surface each day.

Six weeks after the meniscectomy, conventional radiography was performed to confirm low-grade OA, and then the sheep were divided into three groups. One medical doctor responsible for the randomization procedure. The first group (G1; n = 6) received thrice 1 mL of intra-articular exosome Ad-MSC injection at the sixth, seventh, and eighth week after meniscectomy; the second group (G2; n = 6) received twice (sixth and seventh week after meniscectomy) intraarticular injection of 1 mL hyaluronic acid (Durolane™), which is a high-viscosity hyaluronic acid (HA) containing 20 mg of sodium hyaluronate, while the third group (G3; n = 6) received combination of both with the interval of one week for each injection (HA at the 6 ^th^, 7 ^th^, and 8 ^th^; while exosome at the 6 ^th^ and 7 ^th^). The Ad-MSC exosome was prepared using the protocol mentioned in the previous study. ^18^

Each subject was euthanized at the end of the 12 ^th^ week after the radiological evaluation. Before the sacrifice, the anesthesia procedure was the same as the meniscectomy procedure. The euthanasia procedure involves dissecting the sheep’s carotid artery until the sheep bled to death. The joint that received the intervention and the contralateral were harvested for evaluation.

### Assessment protocol

Clinical, radiological, and macroscopic observation was performed 12 weeks after injection to evaluate the outcome. Two veterinarians recorded the severity of clinical signs monthly using the clinical lameness score by Nganvongpanit et al. At the same time, one radiologist and one orthopaedic surgeon evaluated the conventional radiographic score by Innes et al. The macroscopic evaluation was performed after the sheep were sacrificed in the 12 ^th^ week using the OARSI score performed by two orthopaedic surgeons. The mean value of each score was obtained and analyzed using SPSS for Mac.

### Clinical lameness score (CLS) ^20^

The effectiveness of the therapy was evaluated using a clinical grading system that evaluated the individual animal’s lameness, palpable discomfort, and ability to support its weight ( Table 1). Two vets assessed the sheeps’ lameness by having them walk and trot 6 meters three times. Palpation of the stifle joint was then performed to assess discomfort and determine joint mobility. Two veterinarians, separated by thirty minutes, conducted the palpation. Following surgery, this assessment was done each month from 10 days, one month, two months, and three months post meniscectomy.

**Table 1.** Clinical lameness score criteria.

Criteria	Grade	Clinical Evaluation
Lameness	1	Walks normally
	2	Slightly lame when walking
	3	Moderately lame when walking
	4	Severely lame when walking
	5	Reluctant to rise and will not walk more than five paces
Pain on palpation	1	None
	2	Mild signs; turns head in recognition
	3	Moderate signs; pulls limb away
	4	Severe signs; vocalizes or becomes aggressive
	5	Will not allow palpation
Weight-bearing	1	Equal on all limbs standing and walking
	2	Normal standing; favors affected limb when walking
	3	Partial weight-bearing standing and walking
	4	Partial weight-bearing standing; non- weight-bearing walking
	5	Non-weight-bearing standing and walking

### Radiographic evaluation

Before the meniscectomy, six weeks, and 12 weeks after the meniscectomy, radiographic examinations were conducted using a high-resolution film screen combination using the POX-100BT by POSKOM, Gyeonggi, Seoul, Korea, and viewed using the VetDROC application by Insan Teknotama Bersahaja. Radiological evaluation was evaluated using conventional radiographic OA score for stifle joints by Innes et al. ^15^ Parametes that evaluated using radiographic OA score for stifle joints by Innes et al. includes gloval score for overall disease severity, joint effusion, osteophytosis, intra-articular mineralization, and tibial subchondral sclerosis. The breakdown of how to score each parameter can be seen in Table 2. The total mean score was used for evaluation. The results are evaluated and scored twice by one orthopedic surgeon and radiologist. The hindlimb stifles joint procedure executes mediolateral and craniocaudal projections of the stifle joints for each sample. In order to achieve a position that appeared to be “weight-bearing” for the craniocaudal projection, the sheep were kept in the “sitting” position. The ovine was placed in lateral decubitus for mediolateral projection, with the contralateral limb held away from the film. Using the craniocaudal and mediolateral hindlimb stifle protocols, Insan Teknotama Bersahaja processed all of the results using the VetDROC application.

**Table 2.** Radiographic OA score for stifle joints. ^15^

Radiographic score	Score
Global score for overall disease severity	0-2
Joint effusion	0-2
Osteophytosis	0-3
Intra-articular mineralization	0-2
Subchondral sclerosis	0-1

### Macroscopic examination

The specimens of the knee joints were sacrificed before macroscopic and microscopic evaluation using the Osteoarthritis Research Society International (OARSI) score. ^16^ The total gross deterioration of the entire articular surface is taken into account by the OARSI scoring system for macroscopic pathology, which evaluates the cartilage, osteophyte, and synovial membrane. ^16^ Macroscopic evaluations were performed at the lateral femoral condyle, lateral tibial plateau, trochlear groove, and patellar region. The visual assessment was performed twice within a one-week interval. Before the examination, macroscopic specimen images were randomized by a general practitioner assistant unaware of the specimen sample codes. Each component for OARSI macroscopic evaluation was presented in Table 3.

**Table 3.** OARSI macroscopic evaluation. ^16^

No	Parameter	Points
**1.**	**Articular cartilage damage**	
	**Cartilage evaluation:**	
	1. Normal.	0
	2. Rough surface.	1
	3. Fibrillation dan fissure.	2
	4. Small erosion to subchondral bone (< 5 mm).	3
	5. Large erosion to subchondral bone (> 5 mm).	4
**2.**	**Osteophyte**	
	**Osteophyte evaluation:**	
	1. Normal.	0
	2. Mild osteophyte formation (size < 2 mm or < 20% joint margin).	1
	3. Moderate osteophyte formation (size 2 – 4 mm or 20-50% joint margin).	2
	4. Major osteophyte formation (size > 4 mm or > 50% joint margin).	3
**3.**	**Synovium characteristics**	
	**Synovium evaluation:**	
	1. Normal – Opal white, semitranslucent, smooth with sparse blood vessels and clear borders.	0
	2. Minimal – Focal involvement, minimal discoloration, minimal thickening/fibrillation, minimal increased vascularity.	1
	3. Mild – Diffuse involvement, minimal discoloration, consistent minimal thickening/fibrillation, moderate increased vascularity.	2
	4. Moderate – Diffuse involvement, moderate discoloration, moderate fibrillation/thickening, moderate increased vascularity.	3
	5. Severe – Diffuse involvement, severe discoloration, severe fibrillation/thickening, diffuse synovial proliferation with diffuse hypervascularity.	4
	6. Profound – Diffuse involvement, severe discoloration, very severe fibrillation/thickening, thickening to fibrosis with proliferation and diffuse hypervascularity.	5

### Microscopic examination

Following the sacrifice, the knee joint specimens were decalcified in an electrolytic decalcifying solution and subsequently preserved in 10% formaldehyde. Following the decalcification process, the most damaged 5 × 5 mm area was carefully removed from each region—the lateral femoral condyle, lateral tibial plateau, trochlear groove, and patellar region—and immersed in paraffin before being sectioned at a thickness of 5 μm. Hematoxylin-eosin and safranin O staining were used for histological staining. Using the ImageJ plugin, Fiji, observations were obtained from multiple low power fields and stitched together to show the entire cartilage surface. Histopathologic OARSI score was evaluated and scored as presented in Table 4.

**Table 4.** OARSI microscopic evaluation. ^18^

Grade	Subgrade
**Grade 0: surface intact, cartilage intact**	No Subgrade
Grade 1: uneven but intact surface Possible features: superficial fibrillation, cell death and proliferation	1.0 cell intact 1.5 cell death
Grade 2: surface discontinuity	2.0 fibrilation through superficial zone 2.5 Superficial abrasion with matrix loss within superficial zone
Grade 3: vertical fissures	3.0 Simple fissures 3.5 Branched/complex fissures
Grade 4: erosion	4.0 Superficial zone delamination 4.5 Mid zone excavation
Grade 5: denudation	5.0 Bone surface intact

### Data collection and statistics

All data was presented descriptively using mean ± SD. Comparison treatment results of each group were analysed using a multivariate ANOVA test with statistically significant results using *p < 0.05.*

## Results

From 18 sheep that were performed total lateral meniscectomy at the right hind limb’s stifle joint, all wounds were completely healed within ten days. There were no side effects after intra-articular injection for the whole group, such as infection, swelling, or death. The mean weight of all sheep was 28.61 ± 5.48; G1 was 29.33 ± 6.06; G2 was 28.00 ± 5.48; and G3 was 25.50 ± 2.59, with p = 0.184, which was statistically not significant.

### Clinical lameness score

All parameters of CLS between groups statistically decreased significantly from day 10, 1 ^st^ month, second month, and third month after meniscectomy ( *p* < 0.0001) ( Table 5). However, there were no significant differences in this decrease between groups. The lameness parameter scored lowest in G3 compared to other groups significantly on the second month (2.0±0.0 VS 2.7±0.52 VS 2.7±0.52); however it did not differ in the following months.

**Table 5.** Clinical lameness score post meniscectomy comparison between groups.

	Group 1	Group 2	Group 3	p value
**Lameness**				
10 days	3.5±0.55	3.5±0.55	3.5±0.55	*p = 1.00*
1 month	2.5±0.55	2.7±0.52	2.5±0.55	*p = 0.87*
2 months	2.7±0.52	2.7±0.52	2.0±0.00	*p = 0.022* **
3 months	2.5±0.55	2.7±0.52	2.3±0.552	*p = 0.561*
**Pain**				
10 days	4.7±0.52	4.5±0.55	4.5±0.55	*p = 0.827*
1 month	3.5±0.55	3.8±0.75	3.8±0.75	*p = 0.701*
2 months	3.8±0.75	3.7±0.82	3.5±0.55	*p = 0.727*
3 months	3.2±0.75	3.2±0.98	3.0±0.89	*p = 0.931*
**Weight bearing**				
10 days	3.3±0.52	3.5±0.55	3.7±0.52	*p = 0.561*
1 month	3.5±0.55	3.7±0.52	3.5±0.55	*p = 0.827*
2 months	3.3±0.52	3.7±0.52	3.3±0.52	*p = 0.454*
3 months	2.5±0.55	2.7±0.52	2.5±0.55	*p = 0.827*
**Total**				
10 days	11.5±0.55	11.5±0.55	11.67±0.52	*p = 0.827*
1 month	9.5±0.84	10.0±0.89	10.0±0.63	*p = 0.472*
2 months	9.83±1.17	9.5±0.84	9.33±1.03	*p = 0.695*
3 months	8.17±0.75	8.33±1.03	8.0±1.27	*p = 0.858*

Group 1 = exosome 3x; Group 2 = HA 2x; Group 3 = combination exosome 3x and HA 2x.

* Data presented in mean ± SD.

** Statistically significant result with *p value < 0.005*.

### Radiologic evaluation

**Figure 1.**
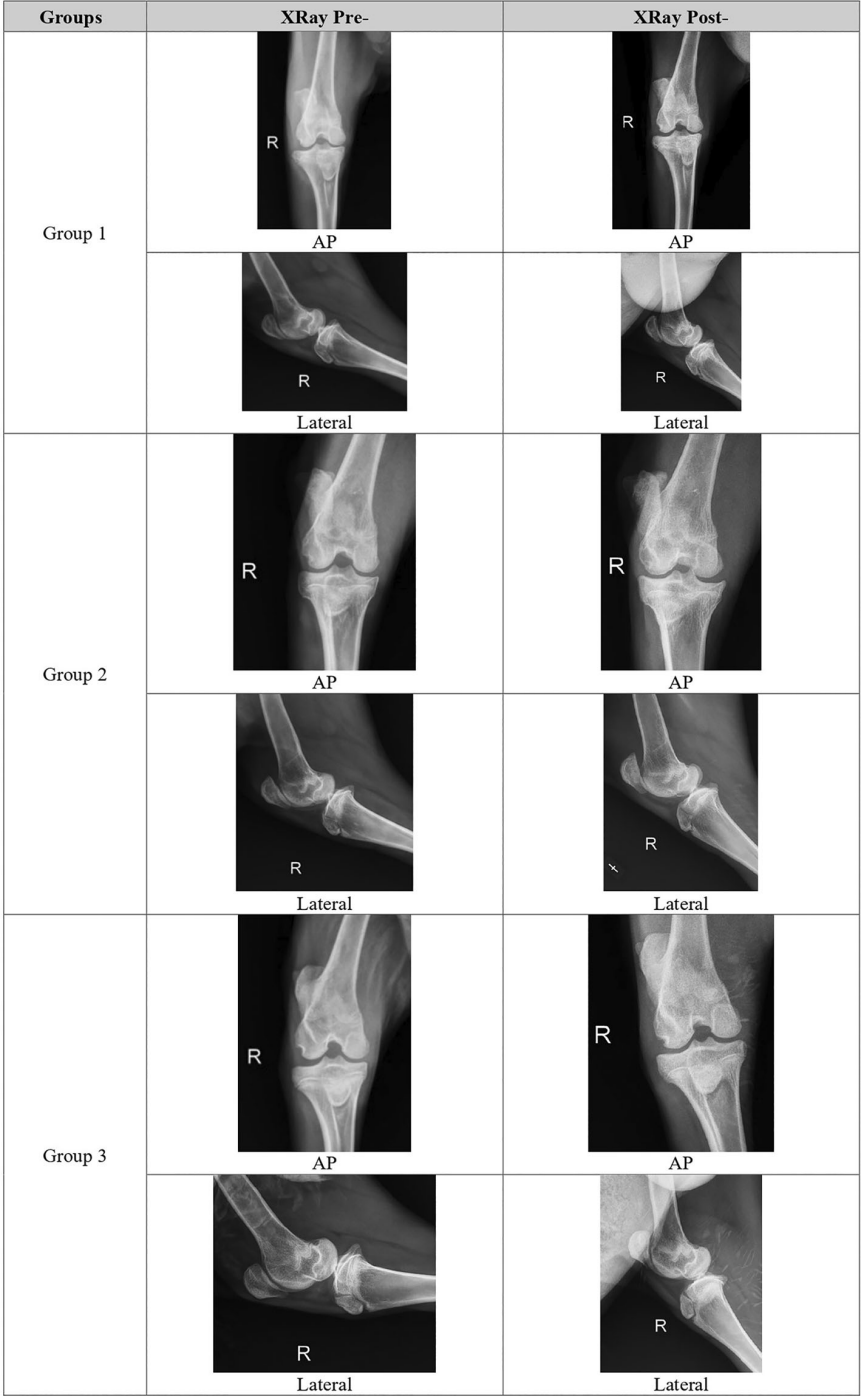
Representative of each group for radiological evaluation.

Radiologic score was increased 18 weeks vs 6 weeks after meniscectomy in the G1 and G2, but decreased although not significantly in the G3. The combination group (G3) followed with G1 and G2, had the lowest total OA radiographic score compared to all groups (5.2±1.17 vs 6.3±0.82 vs 6.7±1.03), *p = 0.053.* Each group representative for radiological evaluation can be seen in Figure 1.

Global severity in G1 and G2 increased but not in G3, and this result was statistically significant ( *p = 0.024*). Meanwhile, effusion score in the 18 ^th^ week reduced significantly in all groups. In contrast, global severity of the disease, osteophyte, and sclerosis score tend to increase, except in G3, where global severity and sclerosis remained the same ( Table 6).

**Table 6.** Radiographic score post meniscectomy comparison between groups.

	Group 1 *	Group 2 *	Group 3 *	p value
**Global**				
6 weeks	1.7±0.52	2.2±0.41	1.7±0.52	*p = 0.152*
18 weeks	2.5±5.5	2.5±0.55	1.7±0.52	*p = 0.024* **
**Effusion**				
6 weeks	2.0±0	1.8±0.41	2.0±0	*p = 0.391*
18 weeks	1.0±0	1.5±0.55	1.7±0.52	*p = 0.046* **
**Osteophyte**				
6 weeks	1.5±0.55	2.0±0.63	1.7±0.52	*p = 0.327*
18 weeks	2.3±0.52	2.2±0.41	1.8±0.75	*p = 0.338*
**Intra-articular mineralization**				
6 weeks	0	0	0	-
18 weeks	0	0	0	-
**Sclerosis**				
6 weeks	0	0	0	*-*
18 weeks	0.5±0.55	0.5±0.55	0	*p = 0.116*
**Total**				
6 weeks	5.2±0.98	6.0±1.10	5.3±0.82	*p = 0.319*
18 weeks	6.3±0.82	6.7±1.03	5.2±1.17	*p = 0.053*

Group 1 = exosome 3x; Group 2 = HA 2x; Group 3 = combination exosome 3x and HA 2x.

* Data presented in mean ± SD.

** Statistically significant result with *p value < 0.005*.

### Macroscopic evaluation by OARSI score

**Figure 2.**
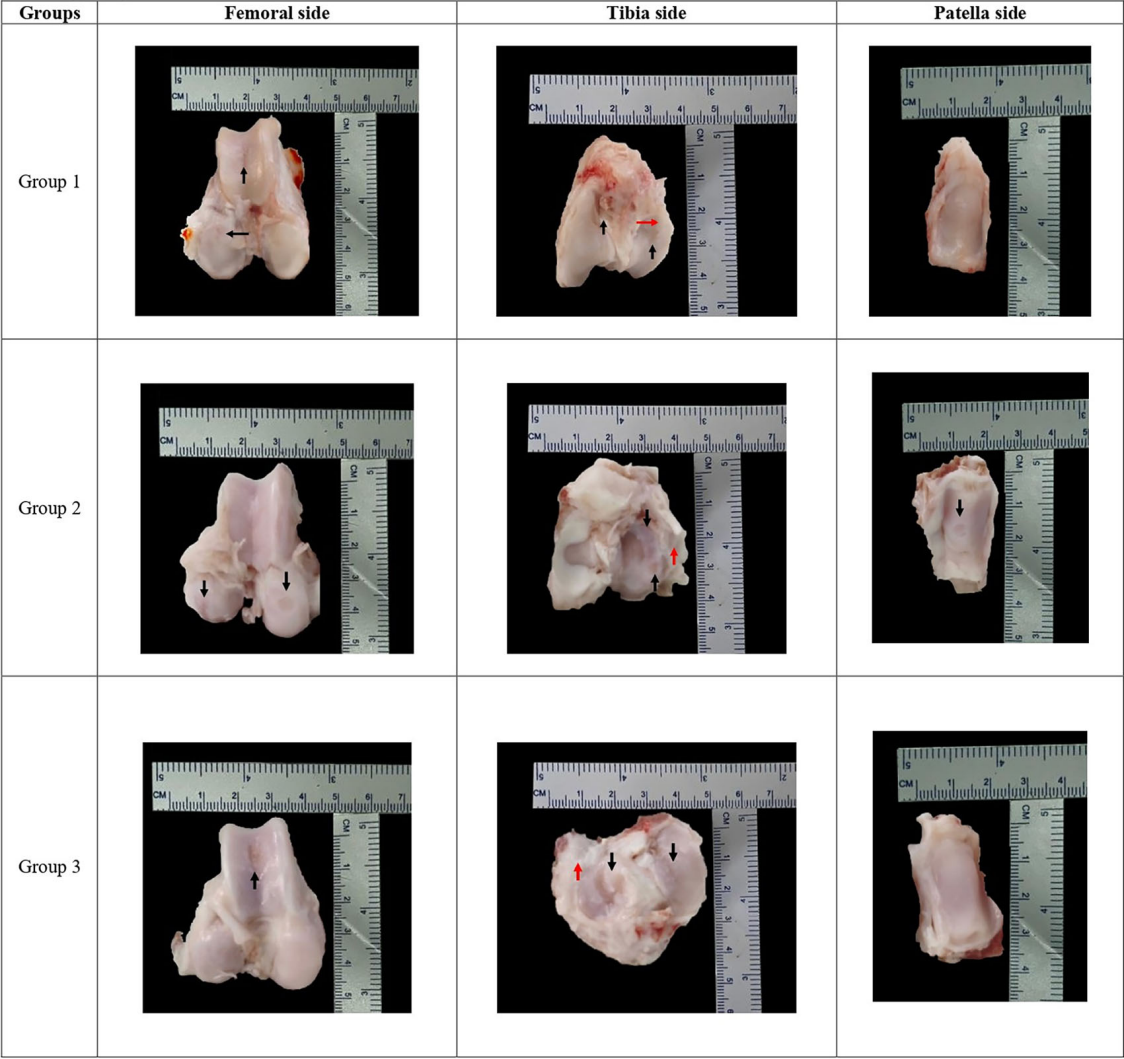
Each group macroscopic findings representative with scale bar.

Mean macroscopic OARSI score evaluation showed no significant differences between groups (G1 9.17 ± 2.32 vs G2 9.83 ± 0.41 vs G3 9.33 ± 0.82); *p = 0.711* ( Table 7). Each group representative for macroscopic finding can be seen in Figure 2.

**Table 7.** Macroscopic OARSI score post meniscectomy comparison between groups.

	Group 1 *	Group 2 *	Group 3 *	p value **
**Cartilage**	3.17 ± 1.72	3.67 ± 0.52	3.83 ± 0.41	0.542
**Osteophyte**	2.83 ± 0.41	2.83 ± 0.41	3.00 ± 0.00	0.616
**Synovium**	3.17 ± 1.72	2.83 ± 0.41	3.00 ± 0.00	0.255
**Total**	9.17 ± 2.32	9.83 ± 0.41	9.33 ± 0.82);	0.711

Group 1 = exosome 3x; Group 2 = HA 2x; Group 3 = combination exosome 3x and HA 2x.

* Data presented in mean ± SD.

** Statistically significant result with *p value < 0.005*.

### Microscopic evaluation by OARSI score

**Figure 3.**
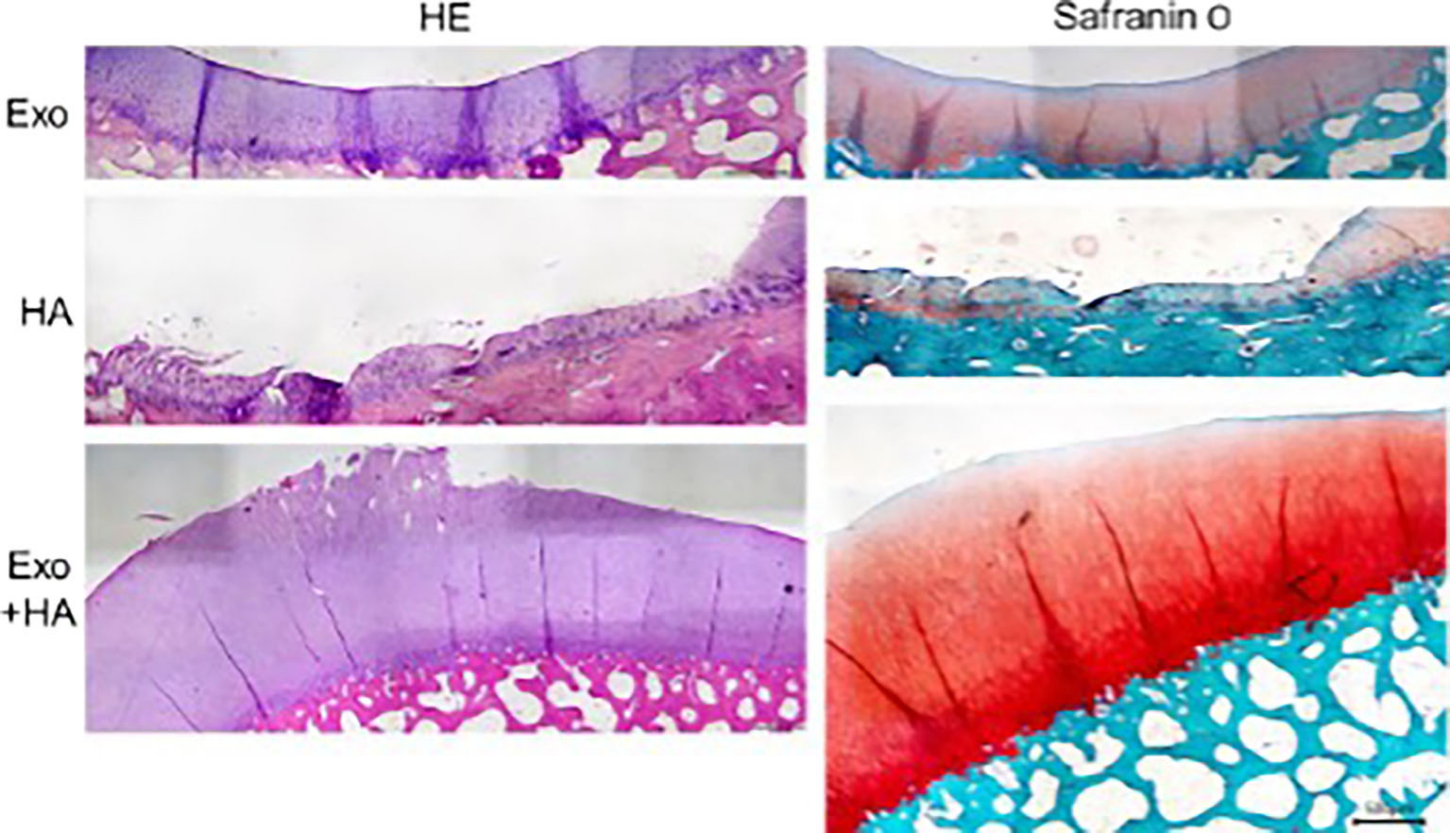
Each group histologic finding representatives with scale bar.

Histopathologic evaluation showed worst grade on the HA group which showed lesion up to middle and deep zone. There were clusters of chondrocytes and sign of hypercellularity, matrix staining was also inhomogeneous while exosome group showed superficial up to middle zone lesion only. The combination group (G3) showed the best microscopic result shown by superficial lesion and few samples had intact cartilage surface as seen in Figure 3.

**Figure 4.**
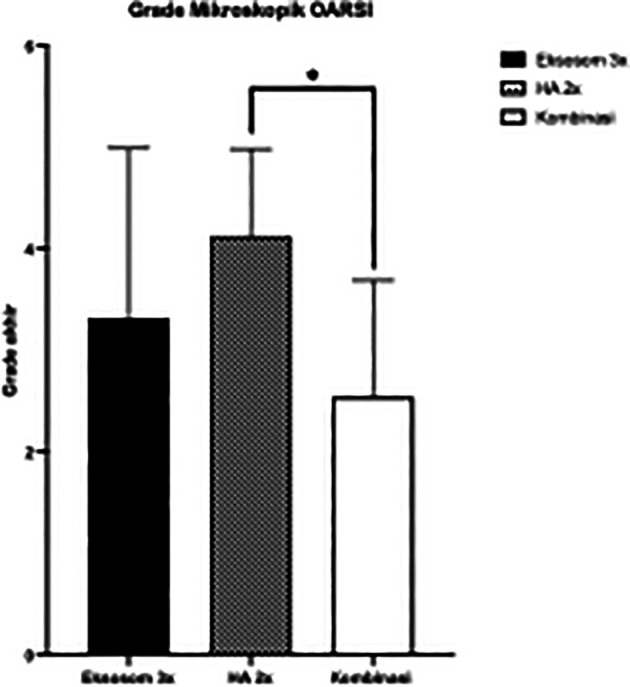
Microscopic OARSI Grade between Groups

Statistical evaluation showed significantly low histopathologic grade in combination group compared to HA only group ( Figure 4).

## Discussion

The most significant finding in this study arises from the clinical, radiological, and macroscopic results. The combined repeated intra-articular injection of exosome Ad-MSC and HA demonstrates superior efficacy in significantly delaying the progression of early OA compared to exosome Ad-MSC alone or HA alone.

We evaluated the clinical signs and symptoms using a Clinical Lameness Score (CLS) on the 10th day, first month, second month, and third month post-operatively. On the 10th day after meniscectomy surgery, all sheep exhibited marked pain and moderate lameness with partial weight-bearing, without significant differences between groups, attributed to post-surgery pain. By the first month, there was a notable improvement in clinical symptoms, with most sheep displaying limping during walking, moderate pain, and partial weight-bearing when standing and walking. Up to this point, no significant differences were observed, reflecting the initial stages of soft tissue healing. However, in the second month, a clinically significant difference in lameness scores emerged, particularly in G3 compared to G2 and G1 (2.0 ± 0.00 vs. 2.7 ± 0.52 vs. 2.7 ± 0.52; *p* = 0.022). Meanwhile, other parameters, such as pain and weight-bearing scores, generally decreased over time.

The problem with cartilage healing lies in its limited ability to heal, attributed to its characteristics as a differentiated tissue with a densely structured extracellular matrix (ECM), avascular, aneural, and alymphatic properties. Therefore, current orthobiologic studies primarily focus on regenerative biological therapy that can, at least, halt and, ideally, reduce the progression of OA. ^12^

The existing evidence suggests that intra-articular administration of MSCs is effective in osteoarthritis treatment. However, there are notable limitations associated with MSC transplantation, including encompassing ethical concerns, the culture process, and production costs, which the metabolite products of MSCs have further elucidated. Ex-vivo tests have indicated that cultivated MSCs exhibit cytotoxicity to osteoarthritic synovial fluid. ^8^ The MSC secretome contains bioactive signals, growth factors, and extracellular matrix (ECM) molecules, with growth factors supporting chondrogenesis. Numerous studies have demonstrated the protective effects of exosomes in osteoarthritis animal models. ^3^^,^^21^ Additionally, higher levels of chondrogenic markers, such as β-catenin and collagen type II, indicate their role in promoting chondrogenesis. ^22^ Recent research by Pye et al., 2022, has explored the progression of canine osteoarthritis, proposing treatment with biologically active signaling molecules found in EVs derived from MSCs. ^23^

Repeated intraarticular injection of HA has been proven to delay human OA progression. ^13^^,^^14^ Our previous study has proven that twice HA injections at one-week intervals are enough to create the protective effect of OA since thrice injections showed no significant result in short-term follow-up (3 months). This study showed that the combination of HA and exosome injection showed the slowest increase in global severity of disease compared to exosome alone or HA alone in the third month (1.7±0.52 vs. 2.5±0.55 vs. 2.5±0.55; *p = 0.024*). However, the effusion parameter was most marked in the G1 vs. G2 and G3 (1.0±0.0 vs. 1.5±0.55 vs. 1.7±0.52; *p = 0.046*). This could be due to the side effect of HA injection, which was creating slight knee swelling, while the exosome has an anti-inflammatory effect and reduced effusion. When co-cultured with activated synovial fibroblasts, exosomes isolated from adipose-derived MSCs upregulated the expression of the anti-inflammatory cytokine IL-10 and downregulated the pro-inflammatory markers such as IL-6, tumor necrosis factor- (TNF-), and nuclear factor kappa B (NF-κB) (Zhao et al., 2020). ^24^

Lubis et al., 2023 showed that compared to hyaluronic acid, intra-articular injection of secretome is beneficial in treating early-stage osteoarthritis in animal models. ^19^ However, this study did not evaluate the combination of effects while the secretome injected was only once. Although the cause of sodium hyaluronate’s ability to reduce pain is not fully known, it may be related to how it affects nerve impulses and sensitivity. ^25^ A decrease in the sensitivity to mechanical stresses of stretch-activated channels found in the membrane of joint mechanonociceptors is correlated with sodium hyaluronate’s analgesic action. ^26^

The impact of sodium hyaluronate on substance P, a tiny peptide involved in pain signal transmission, represents a potential mechanism through which the compound may alleviate pain. It has been demonstrated that sodium hyaluronate inhibits substance P’s induction of enhanced vascular permeability. Additionally, a previous review ^27^ highlighted that sodium hyaluronate significantly influences inflammatory mediators, including prostaglandin E2 (PGE2) and leukotriene levels, tumor necrosis factor-α (TNF-α) production, arachidonic acid release, and nitric oxide (NO) production. Lowering these inflammatory mediators may reduce pain in individuals who receive sodium hyaluronate injections.

Our study demonstrated that the combination of AD-MSCs exosomes and HA injections resulted in the best outcome. Macroscopic evaluation showed lowest OARSI score in combination group however not significant. Meanwhile, histological evaluation showed significantly lowest OARSI score in the G3 compared to HA only group.

A previous study by Salamanna et al. (2019: 872) showed that HA injections were able to improve repair and protect cartilage by inhibiting MMP-3 and MMP-13. ^11^ Cartilage repair by treatment using HA alone was also observed in the study by Wong et al. (2020: 2226) ^3^ using rabbit osteochondral defect model. However, the repair was not sustainable as the gross observation scores worsened after six weeks of observation. This previous study also found that a combination of MSC exosomes and HA resulted in a sustainable cartilage repair, which was shown by improvements in gross observation scores. This finding is in line with our study, which demonstrated better outcomes from the exosomes and HA combination-treated group than those treated with HA alone. However, the previous study used human embryonic stem cell-derived MSC exosomes, whereas AD-MSCs exosomes were used in our study. Exosomes of AD-MSCs have been described to be able to support cell migration, proliferation, and chondrogenic differentiation better than exosomes from bone marrow MSCs or synovium MSCs (Li et al. 2021: 253). Moreover, AD-MSCs are more accesible to harvest, such as through subcutaneous lipoaspiration, and have fewer ethical issues compared to human embryonic stem cells (Miana & González 2018: 2). ^28^

Our study also found that the group treated with exosomes had the worst outcome compared to other groups. A possible explanation of these findings could be that AD-MSCs exosomes require HA to inhibit cartilage degradation while the exosomes stimulate cell migration, proliferation, differentiation, and matrix synthesis (Li et al. 2021: 259). ^21^ Nevertheless, HA alone is also found to be not sufficient as it requires exosomes to result in sustainable cartilage improvement (Wong et al. 2020: 2226). ^3^ Exosome while widely known to have regenerative potential to cartilage has a weakness that is a fast half-life of approximately hours of injection especially due to its lower viscosity and mode of implantation makes it easily degraded. When combined with HA with a higher viscosity, some of the injected exosome will be entrapped inside the knee joint and hence will create a synergistic effect with HA creating a significant changes in the cartilage regeneration.

The main limitation of this study was no control group due to financial limitations. However, we include groups treated with HA and exosome as a direct comparison with the experimented group. Other limitations includes longer follow-ups needed to further evaluate the long-term outcomes and side effects of the treatment. Nevertheless, until the end of the research, there were no significant side effects observed in the treated groups.

In conclusion, intra-articular injection of combined exosome Ad-MSC and HA with an interval of 1 week is proven to delay the progression of OA clinically, radiologically, and macroscopically between 3 months. However, a longer follow-up duration is required to evaluate the longer-term effect.

## Ethics and consent

The institutional review board from Fakultas Kedokteran Universitas Indonesia approved the study protocol, with reference number KET-932/UN2.F1/ETIK/PPM.00.02/2022. We performed the animal studies after receiving approval from the Institutional Animal Care and Use Committee (IACUC) in the School of Veterinary Medicine and Biomedical Sciences at Institut Pertanian Bogor (IPB) University, with reference number 032/KEH/SKE/IX/2022.

## Data Availability

Figshare: SPSS Data for ‘Combined Exosome of Adipose-Derived Mesenchymal Stem Cell and Hyaluronic Acid Delays Early Osteoarthritis Progression of Ovine Sheep Model: Clinical, Radiographic and Macroscopic Evaluation’, https://www.doi.org/10.6084/m9.figshare.25010012. ^29^ Figshare: ARRIVE checklist for ‘Combined exosome of adipose-derived mesenchymal stem cell and hyaluronic acid delays early osteoarthritis progression of ovine sheep model: Clinical, radiographic and macroscopic evaluation’, https://www.doi.org/10.6084/m9.figshare.25487206. ^30^ Data are available under the terms of the Creative Commons Attribution 4.0 International license (CC-BY 4.0).
