# Supplementary material for: Combined exosome of adipose-derived mesenchymal stem cell and hyaluronic acid delays early osteoarthritis progression of ovine sheep model: Clinical, radiographic, macroscopic and microscopic evaluation
Source: F1000Res. 2025 Feb 13;13:494. Originally published 2024 May 17. [Version 3] doi: 10.12688/f1000research.147309.3 (PMC11842963; doi:10.12688/f1000research.147309.3)
Supplement: Peer review history

# Reviewer report: Reviewer response for version 3

**DOI:** 10.5256/f1000research.178040.r366355

**Published:** 2025-02-20

**Authors:** Sree Samanvitha Kuppa

**Affiliations:** 1 Chonnam National University Medical School, Hwasun, South Korea

Dear Authors,

 Thank for the detailed response. I am satisfied with the response to the review comments.

 Thank You

Is the work clearly and accurately presented and does it cite the current literature?

No

If applicable, is the statistical analysis and its interpretation appropriate?

Partly

Are all the source data underlying the results available to ensure full reproducibility?

No

Is the study design appropriate and is the work technically sound?

Yes

Are the conclusions drawn adequately supported by the results?

Partly

Are sufficient details of methods and analysis provided to allow replication by others?

No

Reviewer Expertise:

Osteoarthritis, Inflammation, Cartilage Regeneration, Cell Biology, Molecular Biology

I confirm that I have read this submission and believe that I have an appropriate level of expertise to confirm that it is of an acceptable scientific standard.

# Reviewer report: Reviewer response for version 2

**DOI:** 10.5256/f1000research.170348.r362207

**Published:** 2025-02-05

**Authors:** Sree Samanvitha Kuppa

**Affiliations:** 1 Chonnam National University Medical School, Hwasun, South Korea

This study explored whether injecting a combination of exosomes from adipose-derived mesenchymal stem cells (Ad-MSCs) and hyaluronic acid (HA) could slow down osteoarthritis (OA) progression in sheep with mild OA. Researchers compared three groups: one receiving exosomes alone, another getting HA alone, and a third receiving both treatments.

 Results showed that the combined treatment group had the best improvement in lameness and X-ray OA scores, suggesting a potential protective effect against OA. However, there was no significant difference in joint surface damage among the groups.

 However, before indexing this paper, the following concerns need to be addressed:

1. The paper does not clearly mention the source of the Ad-MSCs. Were they derived from sheep, humans, or another species? Please specify.
2. The paper mentions that exosomes were checked for sterility and characterized using flow cytometry, but the actual data (including conductivity values) is missing. This needs to be included. Either give as supplementary or add in the main paper as a single figure.
3. How much exosome was obtained per extraction? How many extractions were performed? Was each injection taken from the same batch or different batches?
4.  Why was HA injected twice while exosomes were given three times? Please clarify the rationale behind this dosing schedule.
5. The paper states that 1 mL of exosome was injected, but how much actual exosome content (mg) was in this volume?
6. Why was 1 mL of 20 mg HA chosen? Was this based on previous studies, pilot experiments, or another reason?
7.  The results show that exosome treatment was more effective than HA alone. Why didn’t HA work well individually, but showed improvement when combined with exosomes?
8.  Histology results show mild cartilage regeneration when HA and exosomes were used separately, but a dramatic improvement when combined. If the individual effects were weak, how did their combination lead to such a significant difference? Please provide a scientific explanation.

Clarifying these points will help strengthen the paper and ensure a clearer understanding of the study’s impact.

Is the work clearly and accurately presented and does it cite the current literature?

No

If applicable, is the statistical analysis and its interpretation appropriate?

Partly

Are all the source data underlying the results available to ensure full reproducibility?

No

Is the study design appropriate and is the work technically sound?

Yes

Are the conclusions drawn adequately supported by the results?

Partly

Are sufficient details of methods and analysis provided to allow replication by others?

No

Reviewer Expertise:

Osteoarthritis, Inflammation, Cartilage Regeneration, Cell Biology, Molecular Biology

I confirm that I have read this submission and believe that I have an appropriate level of expertise to confirm that it is of an acceptable scientific standard, however I have significant reservations, as outlined above.

# Response

**Published:** 2025-02-11

**Authors:** Ludwig Andribert Powantia Pontoh

**Affiliations:** Orthopaedic and traumatology, Not Applicable, DKI Jakarta, Jakarta Pusat, Indonesia

First of all we would like to thank the reviewers for the time in understanding our study and pointing out several things that needed to be elaborated more to make our study easily comprehended by the readers. Below are lists of inquiries that were questioned by the reviewer:

1. Ad-MSCs was derived from the cryoprecipitate of human AD-MSC secretome which is stored in the Stem Cell and Tissue Engineering bank facility.
2. The exosomes sterility and characterisation result including conductivity test used in this study has been published in the other paper cited in reference no. 19. We have revised and added information about the characterisation narratively in the result section of this revision document.
3. We did thrice injection of exosome from the same donor source with one week interval hence to maintain its purity and content, we did thrice extraction while keeping the volume limited to 1 ml. However, since there have been no previous publication or data to which protein increase the chondrogenesis process derived from exosome Ad-MSC, we haven’t quantify by the number of protein extracted. After this study was conducted we studied the content of the miRNA playing a major role in the chondrogenesis process and the next step would be to find out whether the more volume used would equal the number of miRNA produced within the exosome and that will be the next project of this study.
4. We have published previous pilot study about this proving that twice weekly of injection of HA is the optimal dose compared to once or thrice, while thrice injection of exosome is optimum compared to once or twice injection in the other journal (Fiolin et al, MJMHS, 2025). Previous studies performed up to 12 injections of exosome, however afterwards the author showed that combining with HA will lower the frequency as higher frequency of Intra-articular injection will create higher risk of infection.
5. This study had not measured the quantity of proteins in the exosome. Recent studies believed that miRNA plays a major role in the chondrogenesis process in intraarticular exosome injection. Therefore, in the next project we planned to quantify 370 human miRNA that were upregulated and downregulated using nanostring and found 3 upregulated mirna and 3 downregulatex mirna with their own pathway significantly enhanced after exosome injection which will be elaborated in another study. We believed as to comparing the total protein contained in the exosome, when we wanted to evaluate the chondrogenesis potency it would be more effective if we quantify the expression of chondrogenic miRNA using qRT-PCR and evaluate whether it will be increased along with the added volume in the next study.
6. 1 ml of 20 mg HA was the quantity of HA performed in the other animal study, and understanding the maximum normal volume of lamb synovial joint would be 1.5 ml. Injecting too much volume hence will create added pressure and discomfort to the being.
7. Several studies have shown HA as a visco-supplement in OA aiming to increase lubrication, when combined with exosome which have chondrogenesis property due to its miRNA content that can enhance cartilage regeneration, the effect will synergistically enhanced. We have added this in the discussion section.
8. Exosome while widely known to have regenerative potential to cartilage has a weakness that is a fast half-life of approximately hours of injection especially its lower viscosity and mode of implantation makes it easily degraded. When combined with HA with a higher viscosity, some of the injected exosome will be entrapped inside the knee joint and hence will create a synergistic effect with HA creating a significant changes in the cartilage regeneration. We have added this in the discussion section.

# Reviewer report: Reviewer response for version 2

**DOI:** 10.5256/f1000research.170348.r319754

**Published:** 2024-09-10

**Authors:** Jiao Jiao Li

**Affiliations:** 1 The University of Sydney, St Leonards, NSW, Australia

The authors have adequately addressed the comments, there are no further comments from this reviewer.

Is the work clearly and accurately presented and does it cite the current literature?

Partly

If applicable, is the statistical analysis and its interpretation appropriate?

Yes

Are all the source data underlying the results available to ensure full reproducibility?

Yes

Is the study design appropriate and is the work technically sound?

Yes

Are the conclusions drawn adequately supported by the results?

Yes

Are sufficient details of methods and analysis provided to allow replication by others?

No

Reviewer Expertise:

Regenerative medicine, osteoarthritis, stem cells/MSCs, extracellular vesicles, animal models

I confirm that I have read this submission and believe that I have an appropriate level of expertise to confirm that it is of an acceptable scientific standard.

# Reviewer report: Reviewer response for version 1

**DOI:** 10.5256/f1000research.161493.r306509

**Published:** 2024-08-10

**Authors:** Asma Abdullah Nurul

**Affiliations:** 1 School of Health Sciences, University of Science Malaysia, Kubang Kerian, Malaysia

The manuscript describes the effects of combination of exosome derived from adipose mesenchymal stem cell and hyaluronic acid delays in early osteoarthritis ovine sheep model. The submitted manuscript is not well-written and should be rewritten based on the comments below:

 - Preparation of exosome and how the dosage was determined should be explained in the method.

 - Normal and control groups must be included in the study. 

 - Considering that the experiment conducted on the animal model is invasive and extensive, I believe this study should incorporate additional technical analyses, such as microCT or MRI evaluations, along with histological analyses.

 - Radiographic and macroscopic result requires detailed explanation and comparison between the study groups.

 - The overall manuscript requires rewrite and English check.

Is the work clearly and accurately presented and does it cite the current literature?

Partly

If applicable, is the statistical analysis and its interpretation appropriate?

I cannot comment. A qualified statistician is required.

Are all the source data underlying the results available to ensure full reproducibility?

No

Is the study design appropriate and is the work technically sound?

No

Are the conclusions drawn adequately supported by the results?

No

Are sufficient details of methods and analysis provided to allow replication by others?

Partly

Reviewer Expertise:

Regenerative medicine; tissue engineering; stem cell

I confirm that I have read this submission and believe that I have an appropriate level of expertise to state that I do not consider it to be of an acceptable scientific standard, for reasons outlined above.

# Response

**Published:** 2024-08-13

**Authors:** Ludwig Andribert Powantia Pontoh

**Affiliations:** Orthopaedic and traumatology, Not Applicable, DKI Jakarta, Jakarta Pusat, Indonesia

**Reviewer comment:** The manuscript describes the effects of combination of exosome derived from adipose mesenchymal stem cell and hyaluronic acid delays in early osteoarthritis ovine sheep model. The submitted manuscript is not well-written and should be rewritten based on the comments below:

 - Preparation of exosome and how the dosage was determined should be explained in the method.

**Author response:** ****A previous study have been published explaining detailed step-by-step technique used to produce the exosome from adipose MSC and cited in this study. However brief summary on exosome production has been added in the text.

**Reviewer comment: **Normal and control groups must be included in the study. **​​​​**

**Author response: **Hyaluronic acid has been regarded as the current standard of therapy for moderate osteoarthritis for over than 3 decades that benefit well in delaying the need of arthroplasty. In this study we regard the HA group as the control group as we have obviously understand that total meniscectomy procedure that we performed will create progressive OA hence needed treatment. On the other hand, we have also performed preliminary study that evaluated the effect of control group with normal saline injection, each 1-3 times injection of HA and exosome to evaluate the preliminary outcome of those groups and showed normal saline group consistently showed worst outcome in all parameter. Hence in this study we only evaluated the group with best outcome and regard the HA group as the control group.

**Reviewer comment: **Considering that the experiment conducted on the animal model is invasive and extensive, I believe this study should incorporate additional technical analyses, such as microCT or MRI evaluations, along with histological analyses.

**Author response:** ****We have also performed MRI evaluations with T2 map value analysis that has been published at another paper cited in this study and histological analysis outcome that has been added in this study in the edited version.

**Reviewer comment: **Radiographic and macroscopic result requires detailed explanation and comparison between the study groups.

**​​​​​​​Author response:** a newer version of this study has been updated to elaborate the results between study groups.

**Reviewer comment:** The overall manuscript requires rewrite and English check.

# Reviewer report: Reviewer response for version 1

**DOI:** 10.5256/f1000research.161493.r282028

**Published:** 2024-06-12

**Authors:** Jiao Jiao Li

**Affiliations:** 1 The University of Sydney, St Leonards, NSW, Australia

This was a brief study on treating osteoarthritis (OA) in an ovine model over 3 months, using adipose MSC-derived extracellular vesicles (EVs), hyaluronic acid, or a combination of the two. The analysis was not extensive and would have benefitted from a longer follow-up as well as control groups, which have been identified as limitations. The value of the study lies in that it is one of the first in the field of testing MSC-EVs in animal models of OA to have used a large animal model (sheep) that would be physiologically representative of human joints and OA disease. The reported lack of a significant improvement over 3 months generally in all of the tested groups is also of value to the field, considering the positive outcomes seen so far in small animals using similar treatment approaches, and highlight the possible challenges in translating an EV-based approach to human OA therapy. More specific comments are provided below.

 - It is suggested to better capture the current status of literature using MSC-derived EVs to treat OA in preclinical models. A sizeable number of studies have been published on this topic, as reflected in recent reviews (Ref1,2,3). Although studies in large animals such as sheep are severely lacking, which gives value to the current study, the background literature and current understanding on the topic should be properly acknowledged in the introduction.

 - It is critical to include the method for isolating and characterising exosomes used for in vivo injection, even if this has been reported previously (ref 19 was cited) – because this is a fundamental aspect of the study. It is necessary to at minimum state the cell source for deriving exosomes, isolation process, and methods of characterisation in accordance with MISEV recommendations (e.g., western blot, nanoparticle tracking analysis, electron microscopy). Moreover, there needs to be a clear statement on the dose of EVs used for in vivo injection (e.g., in particles/mL or amount of protein/mL), and a justification for why this dosage was chosen considering the size of the animal/joint.

 - Following from the above comment, the characterisation data of EVs used for injection need to be reported. At minimum, the study needs to demonstrate that actual EVs were injected (rather than other possibilities e.g., secretome or proteins from the conditioned medium), and the size range/distribution of EVs.

 - It is a limitation that the study groups did not include a MSC injection control with the same number of cells that theoretically provided the dose of EVs. This was alluded to in the limitations as the absence of control groups, but it is recommended to specifically identify the lack of a MSCs-only control (alongside also the lack of an untreated control). Moreover, it would have been interesting to see the histological findings from explanted joints of different groups. The absence of these results for whatever reason should be discussed and identified as one of the limitations.

Is the work clearly and accurately presented and does it cite the current literature?

Partly

If applicable, is the statistical analysis and its interpretation appropriate?

Yes

Are all the source data underlying the results available to ensure full reproducibility?

Yes

Is the study design appropriate and is the work technically sound?

Yes

Are the conclusions drawn adequately supported by the results?

Yes

Are sufficient details of methods and analysis provided to allow replication by others?

No

Reviewer Expertise:

Regenerative medicine, osteoarthritis, stem cells/MSCs, extracellular vesicles, animal models

I confirm that I have read this submission and believe that I have an appropriate level of expertise to confirm that it is of an acceptable scientific standard, however I have significant reservations, as outlined above.

## References

[1] : Current understanding of MSC-derived exosomes in the management of knee osteoarthritis. *Exp Cell Res* .2022;418(2) : 10.1016/j.yexcr.2022.113274 113274 10.1016/j.yexcr.2022.113274 35810774

[2] : Mesenchymal Stem Cell-Derived Exosomes: A Potential Therapeutic Avenue in Knee Osteoarthritis. *Cartilage* .2021;13(1_suppl) : 10.1177/1947603520962567 1572S-1585S 10.1177/1947603520962567 33016114 PMC8808857

[3] : Exosomes derived from MSC as drug system in osteoarthritis therapy. *Front Bioeng Biotechnol* .2024;12: 10.3389/fbioe.2024.1331218 1331218 10.3389/fbioe.2024.1331218 38576449 PMC10993706
